# Automated segmentation of mouse OCT volumes (ASiMOV): Validation & clinical study of a light damage model

**DOI:** 10.1371/journal.pone.0181059

**Published:** 2017-08-17

**Authors:** Bhavna Josephine Antony, Byung-Jin Kim, Andrew Lang, Aaron Carass, Jerry L. Prince, Donald J. Zack

**Affiliations:** 1 Electrical and Computer Engineering, Johns Hopkins University, Baltimore MD 21218 United States of America; 2 Wilmer Eye Institute, Johns Hopkins University School of Medicine, Baltimore MD 21287 United States of America; 3 Department of Neuroscience, The Johns Hopkins University School of Medicine, Baltimore, MD 21287 United States of America; 4 Department of Molecular Biology and Genetics, The Johns Hopkins University School of Medicine, Baltimore, MD 21287 United States of America; 5 Institute of Genetic Medicine, The Johns Hopkins University School of Medicine, Baltimore, MD 21287 United States of America; Justus Liebig Universitat Giessen, GERMANY

## Abstract

The use of spectral-domain optical coherence tomography (SD-OCT) is becoming commonplace for the *in vivo* longitudinal study of murine models of ophthalmic disease. Longitudinal studies, however, generate large quantities of data, the manual analysis of which is very challenging due to the time-consuming nature of generating delineations. Thus, it is of importance that automated algorithms be developed to facilitate accurate and timely analysis of these large datasets. Furthermore, as the models target a variety of diseases, the associated structural changes can also be extremely disparate. For instance, in the light damage (LD) model, which is frequently used to study photoreceptor degeneration, the outer retina appears dramatically different from the normal retina. To address these concerns, we have developed a flexible graph-based algorithm for the automated segmentation of mouse OCT volumes (ASiMOV). This approach incorporates a machine-learning component that can be easily trained for different disease models. To validate ASiMOV, the automated results were compared to manual delineations obtained from three raters on healthy and BALB/cJ mice post LD. It was also used to study a longitudinal LD model, where five control and five LD mice were imaged at four timepoints post LD. The total retinal thickness and the outer retina (comprising the outer nuclear layer, and inner and outer segments of the photoreceptors) were unchanged the day after the LD, but subsequently thinned significantly (*p* < 0.01). The retinal nerve fiber-ganglion cell complex and the inner plexiform layers, however, remained unchanged for the duration of the study.

## Introduction

Optical coherence tomography (OCT) [1, 2] is a noninvasive interferometry-based imaging modality that allows for volumetric imaging of the retina. Since its introduction in 1991, it has found widespread use in the diagnosis and management of a variety of ophthalmic disorders in humans [3–9], as well as in the study of animal models of retinal disease [10–13]. Traditional *ex vivo* histological studies of retinal tissues are capable of visualizing structural and morphological changes in high detail; however, artifacts such as shrinkage and retinal detachment [14–18] are difficult to avoid and present challenges for reproducible quantification. Additionally, due to their nature, *ex vivo* measurements are limited to one time point per eye. To address these limitations, histological studies are now often complemented with spectral-domain OCT (SD-OCT) and other noninvasive imaging modalities such as funduscopy [19] and confocal scanning laser ophthalmoscopy (cSLO) [20]. These technologies, in addition to being noninvasive, also allow for the study of retinal structures in three dimensions (3D).

**Fig 1:**
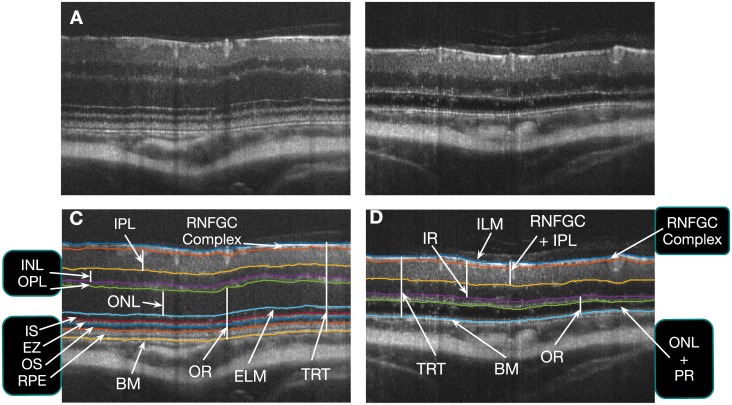
B-scans from (A) a healthy and (B) a light damage (LD) mouse scan. The surfaces segmented in the (C) healthy mouse (10 surfaces), and (D) LD mouse (6 surfaces). The eight retinal layers analyzed in the longitudinal clinical study have also been shown.

Murine models of a variety of ophthalmic disorders have been studied using SD-OCT, where the high-resolution of these images allows for the accurate quantification and tracking of structural changes. For instance, inner retinal thickness changes have been successfully visualized and quantified in rodent models associated with retinal ganglion cell death [21–24]. SD-OCT has also been utilized to visualize outer retinal changes associated with photoreceptor degeneration. Rod degeneration, in particular, has been studied in rd1 and rd10 strains of mice [10, 25, 26]. Another common model of photoreceptor degeneration is the light-induced photoreceptor damage (LD) mouse model [27], which has been previously visualized using SD-OCT [28, 29]. (Fig 1A and 1B show B-scans from a normal and LD mouse scan, respectively.) However, despite the power of SD-OCT to provide highly accurate and temporal *in vivo* imaging of murine retinal structures during the retinal degeneration process, the power of this technology for quantitative analysis of murine models of retinal disease has been underutilized due to the limitations related to the image analysis process. Manual segmentation and measurement of retinal layers of SD-OCT images is time consuming and laborious, and analysis is often limited to layer measurements at single A-scan locations. Moreover, the manual delineations are rater-dependent, and intra- and inter-rater discrepancies cannot be completely eliminated.

Numerous methods have been proposed for the automated segmentation of retinal surfaces in human scans [30–35] and some of these methods have also been extended to animal models [36–38]. In this work, we describe a method for the automated segmentation of mouse OCT volumes (ASiMOV) that is designed to be flexible and easily adapted to a large variety of disease models and scanners. In particular, we address challenges associated with the analysis of large data files (over 400MB) obtained on the high resolution Bioptigen small animal XHR 4110 SD-OCT ophthalmic imaging system (Leica Microsystems Inc., Buffalo Grove IL, USA), while maintaining a reasonable run-time. We also demonstrate the adaptability of the algorithm by applying it to a model characterized by significant structural remodeling. All the tools required for this will also be made freely available at https://www.nitrc.org/projects/aura_tools/ as part of the AURA toolkit.

The paper is organized as follows. We begin with a description of the LD model and the data acquisition, which highlights the motivations behind the flexible strategies incorporated by ASiMOV. This is followed by a description and validation of ASiMOV—the method used to segment healthy as well as LD mouse scans. Next, we demonstrate the use of ASiMOV to segment and analyze retinal layer changes associated with the LD model. Finally, we conclude with a summary of our main contributions and a discussion of our results.

## Mouse model & data acquisition

This section details the LD model, the animals utilized in the experiments, and the acquisition of the SD-OCT scans.

### Mice

Ten female BALB/cJ mice (10—12 weeks) were obtained from Jackson Laboratories (Bar Harbor, ME) and maintained in a cycle of 12 hours of light followed by 12 hours of darkness with optimally controlled temperature and humidity. All experiments were approved by the Institutional Animal Care and Use Committee (IACUC) of Johns Hopkins University School of Medicine and complied with the Association for Research in Vision and Ophthalmology (ARVO) statement for the ethical use of animals in ophthalmic visual research.

### Light damage model

Retinal LD was conducted on five mice as referenced [39]. We chose the inducible LD model to have an advantage to observe a prompt degeneration in outer retinal layer in adult mice. Briefly, the mice were dark-adapted for 24 hours prior to cool-fluorescent light (3000 ∼ 5000 lux) exposure for four hours from 8:00 pm to 12:00 am. Mice were immediately dark-recovered for 12 ∼ 14 hours until the SD-OCT scanning was performed.

### SD-OCT image acquisition

**Fig 2:**
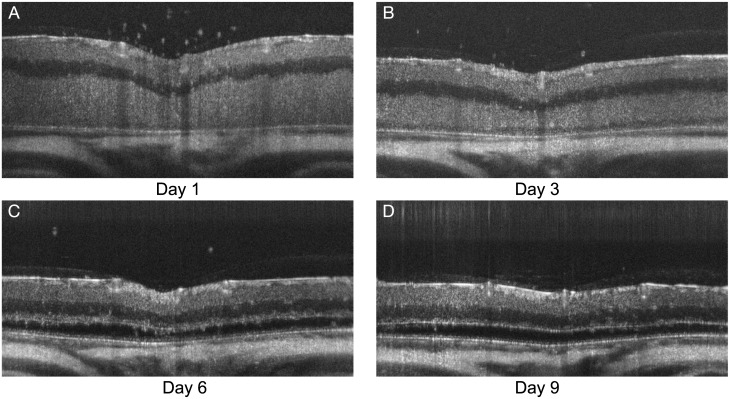
B-scans from an LD mouse (A) 1 day, (B) 3 days, (C) 6 days, and (D) 9 days post light damage.

The mice were first anesthetized using a ketamine/xylazine cocktail (100/10 mg/kg) and then both eyes were imaged using the XHR 4110 SD-OCT ophthalmic imaging system (Leica Microsystems Inc., Buffalo Grove IL, USA). For the image acquisition, the mice were placed in a holding cylinder in order to stabilize fixation and the fundus camera was focused on the optic nerve head (ONH). A rectangular scanning mode (1.4 x 1.4 x 1.6 mm, 1000 A-scans x 100 B-scans with 2048 voxels per A-scan, and a pixel size of 1.4 x 0.78 *μ*m) was used, with ten B-scans acquired at each location. The ten B-scans were averaged in order to improve the signal-to-noise ratio. SD-OCT scans were acquired from both eyes of the mice on four days—1, 3, 6, and 9 days after light-induced retinal damage—giving us a total set of 80 SD-OCT scans. Fig 2 shows examples of the scans obtained at the four time points.

## Methods

ASiMOV segments ten retinal surfaces in healthy mice and six surfaces in LD mice (see Fig 1). In the inner retina, the internal limiting membrane (ILM) and the lower surfaces of the RNFGC complex, inner plexiform layer (IPL), inner nuclear layer (INL), and the outer plexiform layer (OPL) were segmented. In the outer retina, the external limiting membrane (ELM), the bounding surfaces of the ellipsoid zone (EZ) of the photoreceptors, the outer segment (OS) of the photoreceptors, and Bruch’s membrane (BM) were segmented. In the LD mice, however, the photoreceptors are no longer present and therefore only the BM was segmented in the outer retina.

### Automated segmentation of mouse SD-OCT volumes (ASiMOV)

The approach is an adaptation of an existing graph-theoretic approach [32, 33, 36, 37] that allows for the simultaneous segmentation of multiple surfaces that are globally optimal with respect to a cost function. In particular, this method is an extension of the work described by Lang *et al.* [33], which also utilizes a machine learning approach to label voxels in the image as belonging to a particular retinal surface or as background. The use of this machine learning component makes ASiMOV easily adaptable to new models, such as the normal mouse and LD mouse retina.

The SD-OCT scans acquired in this experiment are quite large (100,000 A-scans, ≈400MB), nearly four times larger than a human scan (50,000 A-scans, ≈104MB, Spectralis SD-OCT Scanner, Heidelberg Engineering, Germany). Thus, the processing of these scans present a substantial computational challenge. We addressed this by segmenting the retinal surfaces at a lower resolution, where each B-scan was downsampled by a factor of two in the axial and lateral directions. (Note that the number of B-scans is not altered.) While the graph-theoretic approach can support the simultaneous segmentation of all the surfaces, we segmented the surfaces in three separate steps in order to further reduce both the run-time and memory requirements. In Fig 1C and 1D, the grouped labels indicate that those surfaces were segmented simultaneously.

Before beginning the segmentation, we find the approximate locations of the ILM and the sclera as a preprocessing step. This is done by downsampling the volume further (by a factor of 4) and segmenting the two boundaries simultaneously using the graph-theoretic framework. At this stage the cost functions used consist of gradients in the image. These surfaces were then used to create a region of interest. All subsequent processing is limited within this region of interest.

In the first step, the bounding surfaces of the RNFGC complex were segmented simultaneously. The second step consists of identifying the remaining inner retinal surfaces. In normal mouse scans, the bounding surfaces for the INL and the OPL were segmented simultaneously. However, in the LD scans, only the bounding surfaces of the INL are segmented simultaneously as the lower surfaces of the OPL are not always visible.

Finally, in the third step, the outer retinal surfaces are segmented. In the healthy scans, the ELM, the bounding surfaces of the ellipsoid zone, the retinal pigment epithelium (RPE), and Bruch’s membrane (BM) were segmented simultaneously. Because the photoreceptors are no longer present in the scans from the LD mice, only the top of the ONL and the BM were segmented in this step. As depicted in Fig 2, the ONL may not be visible at certain time points. Nevertheless, six surfaces were segmented in all LD scans, where thickness between top of the ONL and the BM is allowed to reduce to zero.

#### Graph structure & cost function design

The smoothness and interaction constraints that define a feasible set of surfaces were set to fixed expected parameters determined empirically [40]. The smoothness constraints, between B-scans in particular, were relaxed to account for the large motion artifacts often seen in these images. The minimum allowed ONL thickness in the LD mice was also set to zero as this layer is not always visible in the scans (due to edema). Thus, the top of the ONL collapses to the BM when this surface is not visible.

The cost functions of the graph-theoretic approach at each stage were designed using a random forest classifier [41], as detailed by Lang *et al.* [33]. The classifiers were trained on an independent set of scans where manual delineations (BJA) were obtained on five to eight randomly selected slices from six normal and nine LD scans. Note that two separate classifiers were used for the control scans and LD scans, and both were trained on independent sets of annotated data.

## Validation

### Manual delineation

Manual delineations were obtained from three raters (BJA, AC, and AL) on a set of 40 slices. This was done by manually placing control points corresponding to the location of a surface, and then connecting the points with an interpolating spline. Thirty-four volumes were selected at random from the set of eighty. A B-scan to be traced by the raters was then randomly selected from each volume. In order to assess intra-rater variability, a random set of six B-scans was selected from among the 34 and, the locations of these repeated B-scans were randomized and masked from the raters.

The number of surfaces traced varied as certain surfaces (such as the bounding surfaces of the inner and outer segments of the photoreceptors) are no longer present post LD. The interface between the OPL and the ONL is also not always visible immediately after the light-induced damage and, therefore, was only traced on a subset of B-scans.

### Performance analysis of ASiMOV

**Fig 3:**
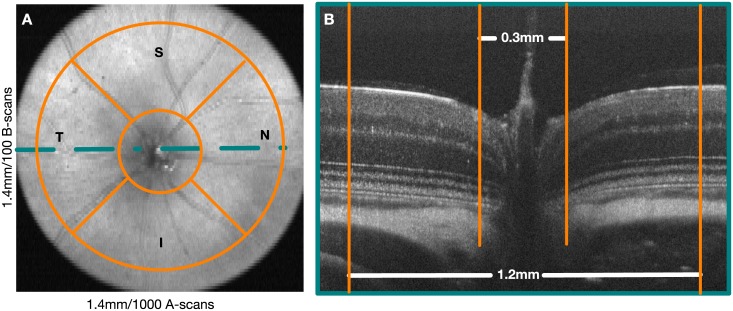
A) En-face image showing the quadrants within which the mean layer thicknesses are assessed. B) A central B-scan, indicating the valid regions that the analysis was limited to. The optic nerve head region and the periphery of the scans are excluded from the analysis.

The SD-OCT scans were centered carefully on the ONH during acquisition. However, the retinal layers are ill defined in this region and thus were omitted from all analyses. A circle 0.3mm in diameter was used to define this region. The periphery was also excluded using a circular mask 1.2mm in diameter. Fig 3 shows the location of the masks in both an en-face image and a B-scan passing through the ONH.

The inter-rater variability was assessed by comparing the raters to each other. The unsigned border position difference (mean ± SD *μ*m) between the delineations obtained from two raters was used as the measure of dissimilarity. The intra-rater variability was computed in a similar manner on the six repeat scans.

The ASiMOV results were compared to the manual delineations obtained from each rater by computing the mean unsigned border position error (mean ± SD *μ*m) for each surface. For the scans where the top of the ONL was not visible and therefore not annotated, the BM is used as a surrogate.

### Results

The intra-rater variability is summarized in Table 1, where the overall variability for all 10 surfaces was observed to be 1.27 ± 0.40 *μ*m, 1.71 ± 0.64 *μ*m and, 1.25 ± 0.48 *μ*m for raters 1, 2, and 3, respectively. Note that only one B-scan from a control mouse was traced twice and thus, no standard deviation numbers are available for the four surfaces that cannot be seen in the LD model. The largest variability was noted for the lower boundary of the RNFGC-complex and the outer segments (OS) of the photoreceptors (≈ 2 *μ*m).

**Table 1:** Intra-rater variability (mean ± SD *μ*m).

Surface	Num. of Slices	Rater 1	Rater 2	Rater 3
ILM	6	0.99 ± 0.38	1.34 ± 0.36	0.66 ± 0.45
RNFGC-IPL	6	2.01 ± 0.41	2.92 ± 1.53	1.52 ± 0.48
IPl-INL	6	1.76 ± 0.41	2.10 ± 1.04	1.57 ± 0.31
INL-OPL	6	1.46 ± 0.37	2.16 ± 0.64	1.45 ± 0.44
OPL-ONL	4	1.26 ± 0.73	2.02 ± 1.18	1.62 ± 0.69
ELM	1	1.25	1.52	1.09
EZ-Top	1	1.00	0.94	0.56
EZ-Bottom	1	1.37	1.10	1.17
OS-RPE	1	0.91	2.00	2.09
BM	6	0.70 ± 0.32	1.00 ± 0.51	0.81 ± 0.29
Average	–	1.27 ± 0.40	1.71 ± 0.64	1.25 ± 0.48

The inter-rater variability is summarized in Table 2, where the mean variability between the raters was found to range from 1.62 to 2.18 *μ*m. The largest variability was noted within the RNFGC-complex, with a range of 2.54 to 3.49 *μ*m. The OPL-ONL boundary was also shown to have higher variability than other surfaces, ranging from 1.77 to 3.45 *μ*m.

**Table 2:** Inter-rater variability (mean ± SD *μ*m).

Surface	Num. of Slices	Rater 1 vs. Rater 2	Rater 1 vs. Rater 3	Rater 2 vs. Rater 3
ILM	34	1.30 ± 0.43	1.00 ± 0.31	1.34 ± 0.38
RNFGC-IPL	34	3.27 ± 1.67	2.54 ± 0.91	3.49 ± 1.39
IPL-INL	34	2.72 ± 0.88	2.19 ± 0.79	3.07 ± 0.85
INL-OPL	34	2.25 ± 0.77	2.45 ± 0.84	2.42 ± 0.93
OPL-ONL	34	2.10 ± 1.08	1.77 ± 0.65	3.45 ± 1.40
ELM	34	1.24 ± 0.48	1.06 ± 0.41	1.29 ± 0.48
EZ-Top	16	1.46 ± 0.38	1.47 ± 0.37	1.29 ± 0.39
EZ-Bottom	16	2.67 ± 0.85	1.11 ± 0.25	2.88 ± 0.73
OS-RPE	16	1.55 ± 0.32	1.64 ± 0.43	1.63 ± 0.48
BM	34	1.06 ± 0.42	0.99 ± 0.40	0.89 ± 0.32
Average	–	1.96 ± 0.42	1.62 ± 0.24	2.18 ± 0.40

**Fig 4:**
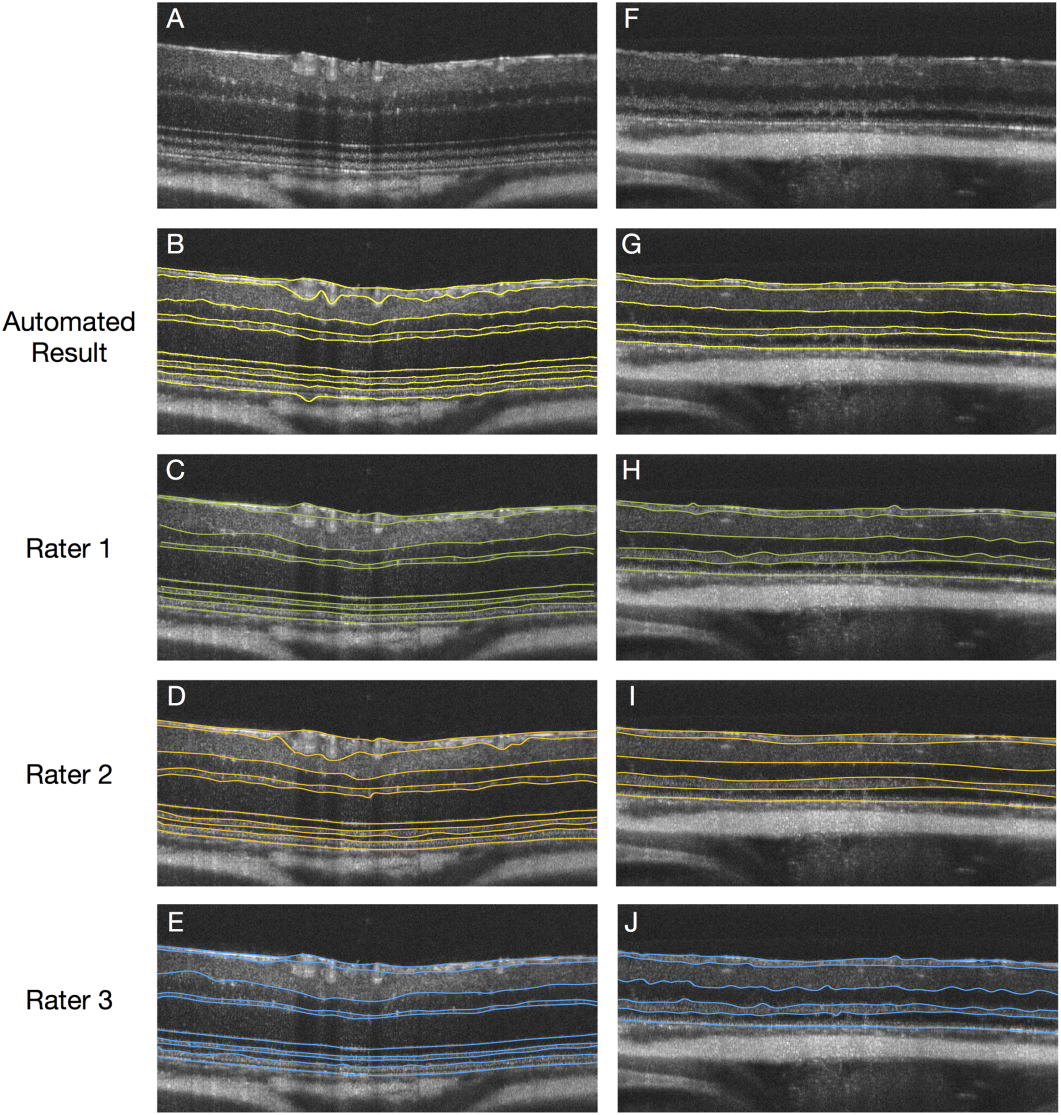
A) B-scan from a control mouse with the B) automated segmentations results overlaid. Manual delineations from (yellow) C) Rater 1 (green), D) Rater 2 (orange) and, E) Rater 3 (blue) are as shown. An example from an LD scan at day 6 is shown in G-J.

The mean unsigned border position error of the automated algorithm is summarized in Table 3. Of the 34 traced slices, 16 came from normal scans and thus, all 10 surfaces were compared to the available manual delineations. Ten slices came from scans where the ONL was not visible and therefore, not annotated. In these slices, the BM was used to represent the top of the ONL. The overall mean error was 1.96 ± 0.61 *μ*m, 2.63 ± 0.94 *μ*m and, 2.14 ± 0.62 *μ*m, respectively, in the normal scans. In the LD scans, the overall error was 2.41 ± 0.56 *μ*m, 2.71 ± 0.73 *μ*m and 2.19 ± 0.49 *μ*m, respectively, which was comparable to the normal scans. Similar to the intra- and inter-rater variability, the largest errors were noted for the lower bounding surfaces of the RNFGC-complex and ranged from 2.88 to 4.61 *μ*m. Fig 4 shows examples of the manual delineations from the three raters as well as the automated segmentation obtained using ASiMOV.

**Table 3:** The mean unsigned border position errors (mean ± SD *μ*m) with respect to three raters.

Surface	Auto vs. Rater 1	Auto vs. Rater 2	Auto vs. Rater 3	Auto vs. Rater 1	Auto vs. Rater 2	Auto vs. Rater 3
ILM	1.41 ± 0.41	1.43 ± 0.40	1.36 ± 0.32	1.40 ± 0.31	1.39 ± 0.34	1.38 ± 0.42
RNFGC-IPL	3.41 ± 1.70	4.61 ± 2.49	3.41 ± 1.87	3.13 ± 1.10	3.38 ± 1.40	2.88 ± 0.77
IPL-INL	2.49 ± 0.94	3.72 ± 1.60	2.49 ± 0.65	2.45 ± 0.68	3.22 ± 0.99	2.16 ± 0.48
INL-OPL	1.72 ± 0.59	2.13 ± 0.66	2.21 ± 0.75	2.50 ± 0.54	2.70 ± 0.64	2.09 ± 0.61
OPL-ONL	2.06 ± 0.59	2.64 ± 1.29	2.32 ± 0.44	2.45 ± 1.27	3.12 ± 1.35	2.19 ± 1.18
ELM	1.66 ± 0.98	2.06 ± 1.08	1.57 ± 1.00	–	–	–
EZ-Top	1.75 ± 1.11	2.49 ± 1.41	2.59 ± 1.18	–	–	–
EZ-Bottom	1.30 ± 1.24	3.03 ± 1.58	1.46 ± 1.39	–	–	–
OS-RPE	2.10 ± 1.25	2.31 ± 1.46	2.16 ± 1.34	–	–	–
BM	1.71 ± 1.46	1.84 ± 1.51	1.83 ± 1.46	2.52 ± 1.16	2.47 ± 1.14	2.43 ± 1.02
Average	1.96 ± 0.62	2.63 ± 0.94	2.14 ± 0.62	2.41 ± 0.56	2.71 ± 0.73	2.19 ± 0.49

## Retinal thickness changes in the LD model

ASiMOV allows for the automated segmentation of retinal surfaces in SD-OCT volumes and thus, can be used to quickly compute thickness measurements from mouse retinal scans. In this section, we demonstrate its use by applying the method to segment the scans obtained from the right eyes of all the mice at the four time points. The thicknesses of eight retinal layers within the control and LD mice were then statistically compared at each time point. Immunohistochemistry was used to further corroborate our findings.

### Statistical thickness changes in normal and LD mice

The automated segmentation results from the right eyes were used to create thickness maps of the retinal layers. A total of eight retinal layers were analyzed —five layers in the inner retina, two in the outer retina, and the total retinal thickness (TRT) (see Fig 1C and 1D). In the inner retina, the RNFGC complex, the IPL, the INL, the combined RNFGC+IPL and, the inner retina (comprised of the RNFGC, the IPL, and the INL) were analyzed. The ONL and the outer retina (comprised of the outer plexiform layer, the ONL, and the photoreceptor inner and outer segments) were also computed and analyzed. Note that in the LD mice, the ONL is defined from the top of the ONL to the BM. The mean layer thickness was assessed within four quadrants, namely the superior (S), inferior (I), nasal (N) and, temporal (T) quadrants bounded by two circles, 0.3 and 1.2 mm in diameter as shown in Fig 3.

In addition to the visualization of the retinal layer thicknesses, the mean thickness within each quadrant of the controls was statistically compared to those obtained from the LD mice at days 1, 3, 6, and 9 post light exposure using paired t-tests. A *p*-value of 0.01 was chosen as a significance test for all tests conducted. The mean layer thickness of the control mice at days 3, 6, and 9 were also compared to those obtained at day 1.

**Fig 5:**
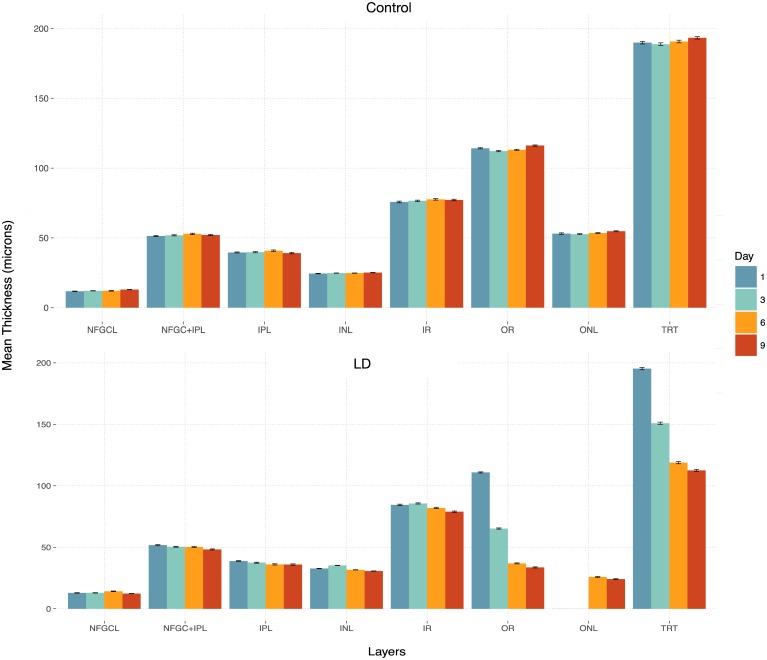
Bar plots of the mean thicknesses over four time points computed in the right eyes of the control (top) and LD mice (bottom).

**Fig 6:**
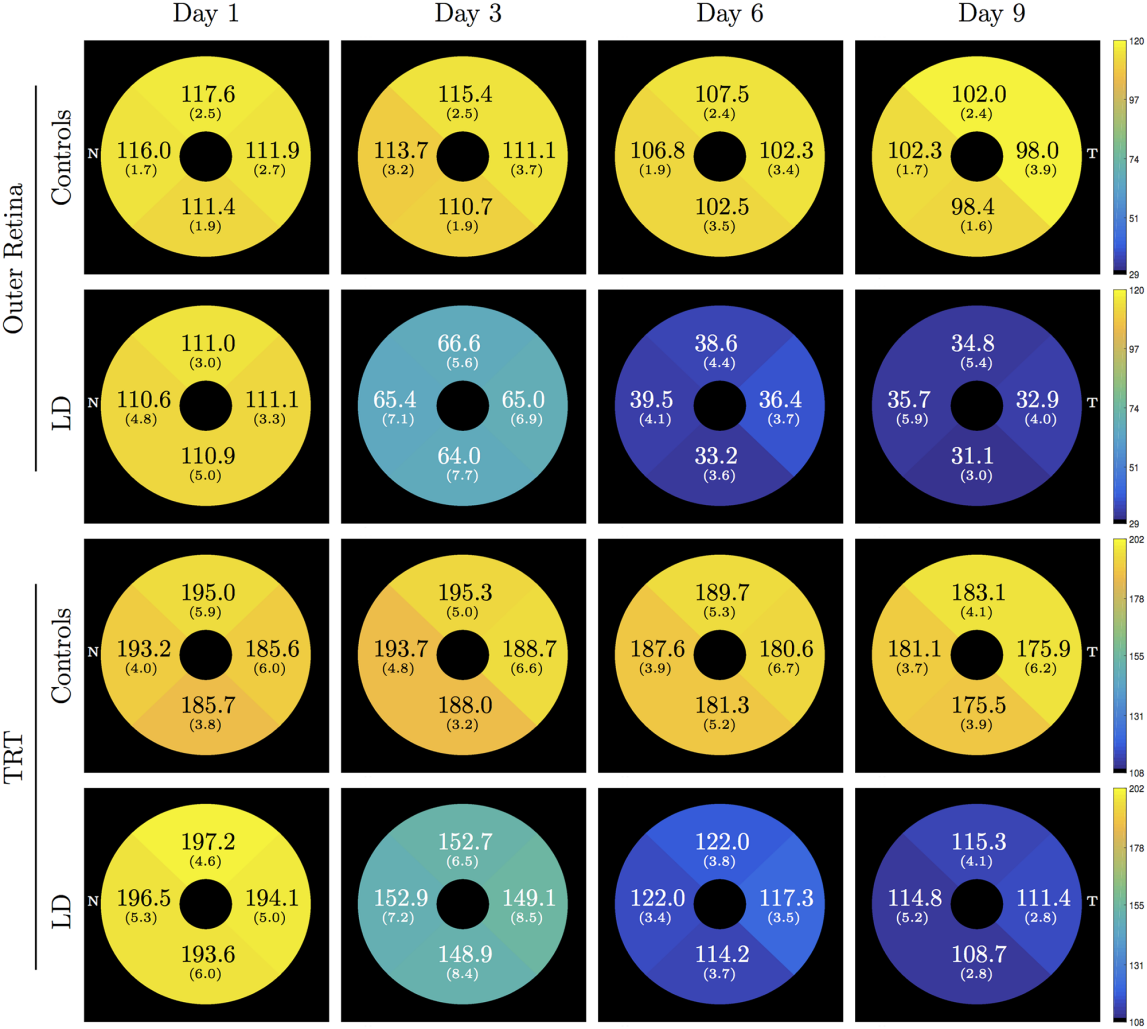
Mean (SD) thickness of the outer retina and the total retinal thickness (TRT) computed for the control and LD mice.

Table 4 summarizes the average thicknesses observed in each of the eight layers for the control and LD mice. For ease of interpretation, the same has also been plotted in Fig 5. Significant differences (indicated by * in Table 4) were noted within the outer retinal layer as well as in the TRT at day 3, 6, and 9. The longitudinal thickness maps of the control and LD mice within the outer retinal layer and the TRT are shown in Fig 6. The mean and standard deviation (*μ*m) thickness computed within each quadrant have been overlaid.

**Table 4:** Summary of average retinal layer thickness observed in the left eyes of control and LD mice. Thicknesses are reported in mean ± SD *μ*m, statistical significance (*p* < 0.01) indicated using *.

Layer	Disease Status	Day 1	Day 3	Day 6	Day 9
RNFGC Complex	Control	11.75 ± 1.02	12.17 ± 0.63	12.60 ± 1.05	12.78 ± 0.53
	LD^1^	12.93 ± 1.10	12.93 ± 0.72	14.23 ± 1.68	12.30 ± 1.77
RNFGC + IPL	Control	51.30 ± 2.25	51.83 ± 1.93	52.89 ± 2.52	52.05 ± 2.18
	LD	51.75 ± 0.98	50.44 ± 1.46	50.31 ± 1.11	48.24 ± 0.55
IPL	Control	39.56 ± 1.64	39.73 ± 1.99	40.81 ± 2.56	39.10 ± 2.60
	LD	38.82 ± 0.84	37.50 ± 1.37	36.08 ± 1.37	35.94 ± 1.46
INL	Control	24.40 ± 0.83	24.75 ± 0.59	24.75 ± 0.49	25.11 ± 0.73
	LD	32.70 ± 0.90*	35.22 ± 0.71*	31.66 ± 1.14*	30.68 ± 0.90*
IR	Control	75.71 ± 3.06	76.58 ± 2.37	77.64 ± 2.87	77.16 ± 2.86
	LD	84.44 ± 1.73*	85.66 ± 2.08*	81.98 ± 1.52*	78.93 ± 1.28
ONL	Control	53.09 ± 2.42	52.63 ± 1.81	53.46 ± 1.80	54.74 ± 1.84
	LD	–	–	25.90 ± 2.97*	24.08 ± 2.41*
OR	Control	114.18 ± 2.10	112.21 ± 2.43	113.08 ± 1.97	116.17 ± 2.10
	LD	110.88 ± 3.87	65.24 ± 6.68*	36.90 ± 3.72*	33.61 ± 4.20*
TRT	Control	189.89 ± 4.85	188.78 ± 4.51	190.72 ± 4.80	193.33 ± 4.32
	LD	195.33 ± 5.08	150.90 ± 7.52*	118.88 ± 3.32*	112.53 ± 3.15*

**Fig 7:**
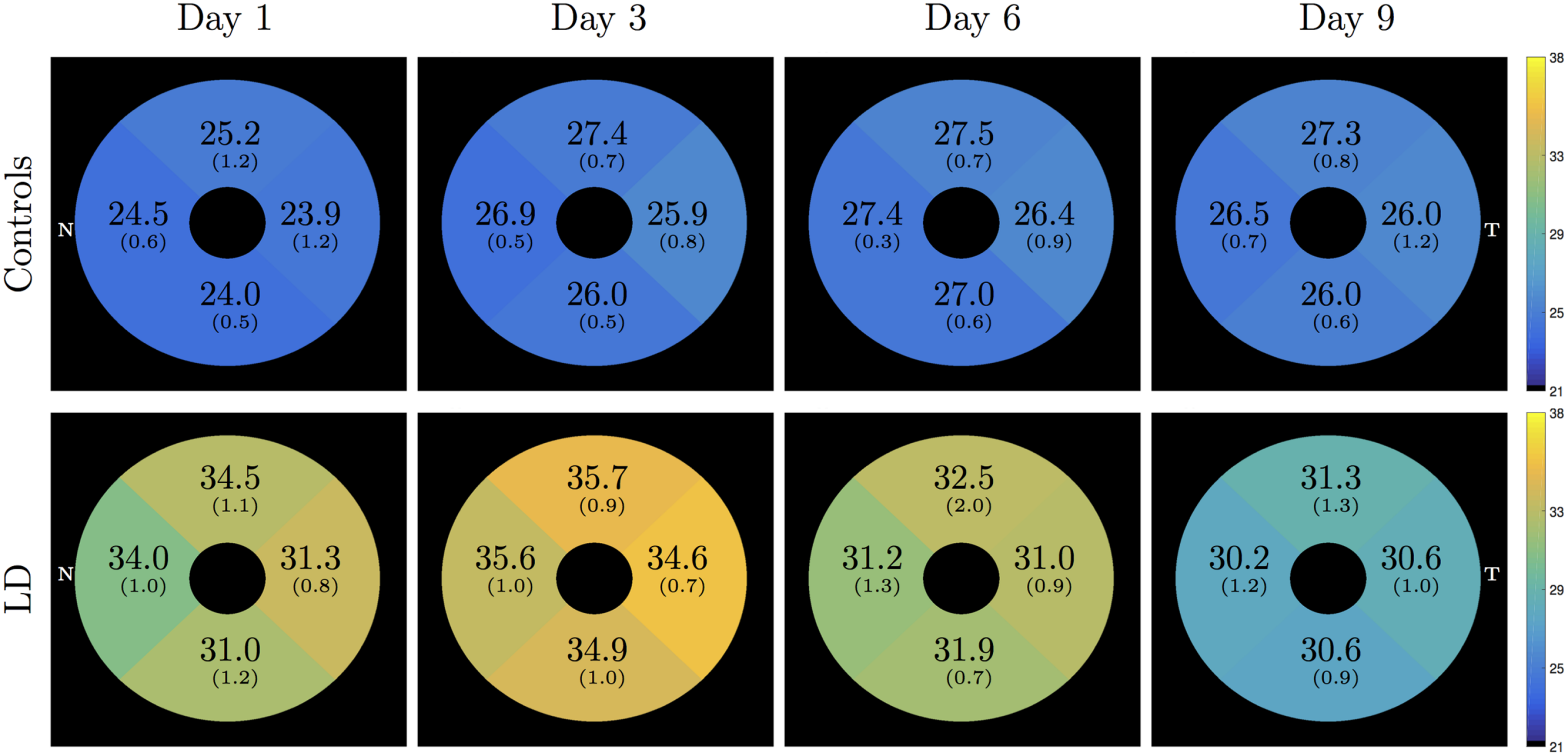
Inner nuclear layer (INL) thickness in the control and LD mice. Mean (SD) thickness is each quadrant was found to be significantly different between the two groups.

A significant increase in thickness was also observed in the INL. Interestingly, this increase was significant at day 1, before a change was detectable in the OR. Fig 7 shows the longitudinal thickness maps of the control and LD mice. All the quadrants showed significantly larger INL thicknesses in comparison to the controls.

The other inner retinal layers, namely the RNFGC complex, the IPL and the combined RNFGC+IPL layers, did not show any differences. The mean layer thicknesses of the control mice on day 3, 6, and 9 did not significantly differ from those obtained at day 1.

### Retinal immunohistochemical analysis

Degeneration of rods and cones was verified by histological analysis of the retinas from the control and LD mice. Retinal immunofluorescence staining for cone arrestin and rhodopsin, and DAPI staining for nuclei, was performed [42]. Eyes were enucleated and fixed in 4% paraformaldehyde and placed in 30% sucrose, followed by embedding in Tissue-Tek O.C.T compound (Sakura Finetek USA, Torrance, CA) for cryosection. Eyes were sectioned at 10 mm thickness on Superfrost Plus microscope slides (Fisher Scientific, Hampton, NH) using a Leica CM3050S cryostat (Leika Biosystems Inc., Buffalo Grove, IL). Slides were washed in PBS and PBST (PBS with 0.1% Triton x100) and blocked for 2 hours at room temperature. Rabbit anti-cone arrestin (ARR) (AB15282, 1:1000, Millipore, Billerica, MA) and mouse anti-rhodopsin (1D4, ThermoFisher scientific, Waltham, MA) were applied to slides at 4°C for 16 hours, followed by incubation for 2 hours at room temperature with anti-rabbit and anti-mouse secondary antibodies, conjugated with Alexaflour 488 and Alexaflour 568 (Invitrogen), respectively. All retinal images were taken using a Zeiss LSM 510 Meta Confocal microscope system.

**Fig 8:**
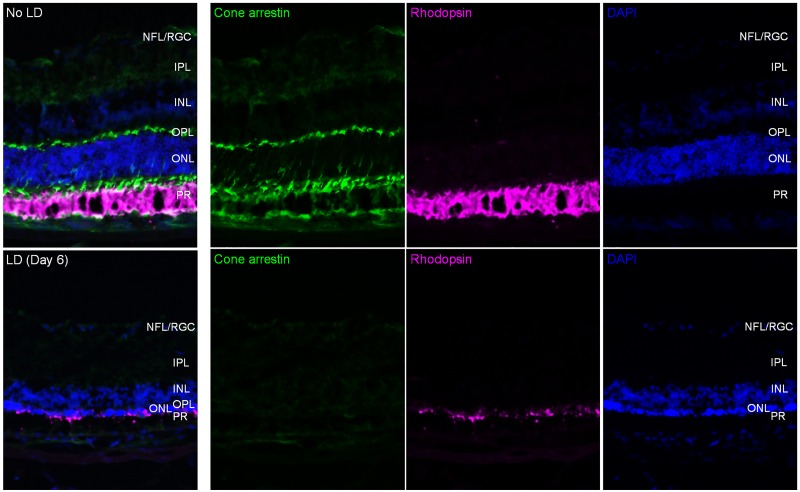
Immunofluorescence for cone arrestin (green) and rod rhodopsin (magenta), and DAPI staining (blue), was imaged from a control mouse eye (top row) and day 6 post LD retina (second row).

Fig 8 shows immunofluorescence images obtained from control and day 6 post LD mouse retinas. As expected, we observed a dramatic decrease of cone arrestin and rhodopsin signals, with a corresponding thinning in the ONL/PR layers in LD retina compared to normal retina, consistent with what we observed through our SD-OCT analysis.

## Discussion

### ASiMOV

The proposed method, ASiMOV, segments ten retinal surfaces in normal mice and six surfaces in LD mice. Comparisons of the method to manual delineations obtained from three raters demonstrated the accuracy and robustness of the proposed method. The largest intra- and inter-rater variability was noted for the outer boundary of the RNFGC-complex and the outer segments of the photoreceptors. These layers are quite thin and difficult to accurately visualize; thus, this variability is not unexpected. The overall unsigned border position difference with respect to the three raters was under 3 *μ*m. Similarly, the largest mean differences were noted in the RNFGC-complex, which was as high as 4 *μ*m. However, this is still comparable with the inter-rater variability. In particular, the blood vessel regions show high variability. Raters 1 and 3 cut through blood vessels while delineating the bottom of the RNFGC-complex but rater 2 didn’t. The random forest was trained using delineations provided by rater 1, however, the large gradients present under the blood vessels encouraged the surface to go around it than through it. The blood vessels present quite a challenge and it is not uncommon [38, 43] to disregard these regions during analysis. In the present work, we have retained the blood vessels, however, in future reproducibility studies we intent to gauge the thickness reproducibility with and without the blood vessel regions.

It is also important to note that the images were downsampled prior to segmentation. Thus, it is possible to further improve the segmentation accuracy, provided the increased run-time can be tolerated. The run-time of the graph formulation utilized here is directly proportional to the size of the graph. Thus, segmenting all the retinal surfaces simultaneously, can be a very memory intensive process that is also slow [32]. However, improvements in run-time can be achieved by using a multi-resolution approach [36, 44] as well as efficient cost function design [33]. Segmenting the volumes at a lower resolution in combination along with efficient cost function design helped limit ASiMOV’s run time to approximately 7 minutes per 3D SD-OCT scan on a quad-core Intel Core i3-4150 3.5 GHz processor with 16GB RAM. This computer was chosen purely for ease of run-time analysis, and is very likely that faster processors will yield results faster.

Previous approaches proposed for the segmentation of mouse retinal surfaces in SD-OCT images have largely worked with scans obtained with a lower resolution and image size [37, 38]. Furthermore, these studies were also conducted using different models. Thus, while direct comparisons cannot be made with these earlier studies, they are similar in certain aspects. The approach proposed by Antony *et al.* [37] utilized the same graph-formulation but did not use machine learning to design the cost function. Srinivasan *et al.* [38] also proposed the use of a graph-theoretic method, albeit a different formulation where the segmentation was posed as a shortest path-finding problem that was solved using dynamic programing. This method was applied to segment the surfaces in a rod degeneration model using rd10 mice, where the photoreceptor layers degenerate over time similar to the LD model. This method does have a key advantage over ASiMOV, in that the same method was used to segment the normal and diseased mice. Theoretically, this can be replicated using the graph formulation proposed in ASiMOV, by relaxing the surface-interaction constraints associated with layers that may disappear in the diseased mice to zero. However, the graph constructed in this case will be unnecessarily large which will likely impact run-time. The training data required for the random forest will also most likely need to be increased to ensure the quality of the cost functions.

### LD model findings

In contrast to the dramatic ONL/PR thinning observed in the LD mice, we consistently observed a slight but significant increase in INL thickness. LD has been purported to cause cellular and molecular changes in the retina, including photoreceptor cell death, increased retinal gliosis, and microglial relocation to the ONL from inner layers [45–47]. However, clear evidence for LD-induced cellular changes in INL cell types (which include bipolar, horizontal, amacrine, Müller, and ganglion cells) in the murine LD model has not been reported. Interestingly, Vihtelic *et al.* [48] showed that LD-induced PR death triggers region-restricted proliferation of Müller cells and INL stem cells in zebra fish, suggesting a potential INL remodeling mechanism after LD. The thickness changes in our data may be associated with a similar process. Defining the exact process of INL thickening will require further research and is beyond the scope of the current study. However, this is crucial to increasing our understanding of retinal reorganization post LD and will be investigated in future studies.

Notably, ASiMOV enabled our new finding with high accuracy and consistency, which is difficult to be obtained from manual thickness delineation using SD-OCT images or microscopic histology analysis after various chemical modification and fixation-related changes in post-mortem retinal tissue.
